# Impacto de la inactividad física en la mortalidad y los costos económicos por defunciones cardiovasculares: evidencia desde Argentina

**DOI:** 10.26633/RPSP.2017.92

**Published:** 2017-07-21

**Authors:** Christian Martín García, José Antonio González-Jurado

**Affiliations:** 1 Pontificia Universidad Católica Argentina Pontificia Universidad Católica Argentina, Cuidad Autónoma de Buenos Aires Argentina Pontificia Universidad Católica Argentina, Cuidad Autónoma de Buenos Aires, Argentina.; 2 Facultad de Ciencias del Deporte Universidad Pablo de Olavide Sevilla España Facultad de Ciencias del Deporte, Universidad Pablo de Olavide, Sevilla, España.

**Keywords:** Actividad física, enfermedades cardiovasculares, mortalidad, valor de la vida, costos de la atención en salud, Argentina, Motor activity, cardiovascular diseases, mortality, value of life, health care costs, Argentina, Atividade motora, doenças cardiovasculares, mortalidade, valor da vida, custos de cuidados de saúde, Argentina

## Abstract

**Objetivo.:**

Estimar la mortalidad y los costos económicos por enfermedades cardiovasculares atribuibles a la inactividad física en Argentina.

**Métodos.:**

Se estimó la mortalidad atribuible (MA) a la inactividad física como el producto entre la fracción atribuible poblacional (FAP) y el número de muertes originadas por las enfermedades cardiovasculares asociadas. Se realizó una valoración del valor estadístico de la vida (VEV) siguiendo el enfoque del capital humano, bajo el cual el VEV se estimó mediante la productividad perdida por muerte prematura. Se calcularon los costos económicos empleando la MA y el VEV, estratificando por sexo, grupo de edad y nivel de actividad física. Se empleó un análisis de sensibilidad para evaluar cómo varían los costos en tres escenarios posibles.

**Resultados.:**

La MA a la actividad física baja y moderada varió entre 33 (18 a 24 años) y 7 857 (> 84 años) defunciones anualmente en ambos sexos. El VEV se encontraba entre I$ 441 005 (dólares internacionales) (18 a 24 años) y I$ 4 121 (> 84 años). La valoración de los costos totales por sexo indica que las pérdidas económicas ascendieron a I$ 752,5 millones para los hombres y a I$ 444,5 millones para las mujeres.

**Conclusión.:**

Las pérdidas económicas variaron entre 0,61% del PIB para el escenario mínimo, 0,85% para el escenario medio, y 1,48% para el escenario máximo. Se recomienda fortalecer el desarrollo de políticas públicas orientadas a la reducción del sedentarismo en Argentina.

## Abstract (en)

**Objective.:**

Estimate mortality and economic costs from cardiovascular diseases attributable to physical inactivity in Argentina.

**Methods.:**

Attributable mortality (AM) from physical inactivity was estimated as the product of the population attributable fraction and the number of deaths caused by associated cardiovascular diseases. Value of statistical life (VSL) was calculated using the human capital approach, in which VSL was estimated through lost productivity from premature death. Economic costs were calculated using AM and VSL, stratifying by sex, age group, and physical activity level. A sensitivity analysis was used to evaluate how costs vary in three possible scenarios.

**Results.:**

AM from low and moderate physical activity ranged from 33 (18 to 24 years) to 7 857 (>84 years) deaths annually in both sexes. VSL ranged from I$441 005 (international dollars) (18 to 24 years) to I$4,121 (>84 years). Assessment of total costs by sex indicates that economic losses amounted to I$752.5 million for men and I$444.5 million for women.

**Conclusion.:**

Economic losses ranged from 0.61% of GDP for the minimum scenario, 0.85% for the average scenario, and 1.48% for the maximum scenario. Stronger public policy-making aimed at reduction of sedentary lifestyles in Argentina is recommended.

## RESUMO

**Objetivo.:**

Estimar a mortalidade e os custos econômicos por doenças cardiovasculares atribuíveis à inatividade física na Argentina.

**Métodos.:**

Foi estimada a mortalidade atribuível à inatividade física como o produto entre a fração atribuível populacional (FAP) e o número de mortes por doenças cardiovasculares associadas. Foi realizada uma avaliação do valor estatístico da vida (VEV) segundo o enfoque do capital humano em que o VEV foi estimado pela produtividade perdida por morte prematura. Foram calculados os custos econômicos empregando a mortalidade atribuível e o VEV, estratificados por sexo, faixa etária e nível de atividade física. Foi feita uma análise de sensibilidade para avaliar a variação dos custos nos três cenários possíveis.

**Resultados.:**

A mortalidade atribuível a um nível baixo e moderado de atividade física variou entre 33 (18 a 24 anos) e 7 857 (>84 anos) mortes ao ano em ambos os sexos. O VEV foi de 441 005 dólares internacionais (18 a 24 anos) a 4 121 dólares internacionais (>84 anos). A avaliação dos custos totais por sexo indicou que as perdas econômicas atingiram 752,5 milhões de dólares internacionais no sexo masculino e 444,5 milhões de dólares internacionais no sexo feminino.

**Conclusão.:**

Os prejuízos econômicos variaram entre 0,61% do PIB no cenário mínimo, 0,85% no cenário intermediário e 1,48% no cenário máximo. Recomenda-se o reforço na elaboração de políticas públicas orientadas à redução do sedentarismo na Argentina.

Según la Organización Mundial de la Salud (OMS), la inactividad física es el cuarto factor de riesgo de mortalidad más importante, a él se le atribuye 5,5% del total de las defunciones a nivel mundial y es responsable de 32 millones de muertes producidas anualmente (1).

Este factor de riesgo se extiende cada vez más por muchos países y repercute en la salud de la población mundial como desencadenante de enfermedades no transmisibles (ENT). Éstas son enfermedades que no se transmiten de persona a persona, son de larga duración, evolucionan lentamente y ocasionan elevados gastos para los sistemas de salud. Entre los diferentes tipos de ENT se encuentran las enfermedades cardiovasculares, la diabetes y el cáncer (2). A nivel global, en 2008 de los 57 millones de defunciones que se produjeron, 63% se debieron a ENT y más de 80% de estas muertes fueron causadas por las enfermedades cardiovasculares y la diabetes (3).

En Argentina, el porcentaje de la población expuesta a la inactividad física continúa en constante aumento. De acuerdo con la Encuesta Nacional de Factores de Riesgo (ENFR) 2005, la prevalencia del nivel de actividad física baja se detectó en 46,2% de la población. En 2009 ascendió a 54,9% y en 2013, a 55,1% (4).

Además de ser un importante factor de riesgo, la inactividad física genera una notable carga económica para el sistema de salud. En un análisis de la inactividad física y la obesidad en el sistema público de salud en la provincia de Ontario, Canadá, en 2009 se estimó que su carga económica se aproximaba a $US 3,5 mil millones ($US 1,2 mil millones en costos directos y $US 2,34 mil millones en costos indirectos) (5). En Australia, se desarrollaron modelos de simulación para calcular cuál sería el beneficio económico que generaría una reducción de 10% de la inactividad física. Los resultados indican que esta reducción se traduciría en 6 000 casos menos de nuevas enfermedades, 2 000 muertes menos, 114 000 días laborales ahorrados y 180 000 días en la producción del hogar, con un ahorro de costos para el sector salud de 96 millones de dólares australianos (6). En los Estados Unidos de América se estimaron los costos económicos relacionados con la atención médica a causa de la sarcopenia (pérdida de masa muscular) sobre una población objetivo de adultos mayores de 60 años. Para el año 2000, los costos directos debidos a la atención médica atribuibles a la sarcopenia ascendieron a $US 18,5 mil millones ($US 10,8 mil millones para los hombres y $US 7,7 mil millones para las mujeres), lo que representaba alrededor de 1,5% del total de los gastos médicos de ese año (7). También hay pruebas científicas sólidas de que los costos directos atribuibles a la inactividad física se encuentran entre 1 y 4% de los gastos totales en el cuidado de la salud y que los costos indirectos superan más del doble de los costos directos (8). Asimismo, varios estudios demuestran que las intervenciones de actividad física son costo-efectivas (9,10).

Aunque en Argentina se han realizado evaluaciones de este tipo con otros factores de riesgo, como, por ejemplo, el tabaco (11), el consumo de sustancias psicoactivas (12) y el sida (13), hasta la fecha no se han estimado los costos de la inactividad física. Por consiguiente, el principal objetivo de este estudio es estimar la mortalidad por enfermedades cardiovasculares atribuible a la inactividad física en Argentina en 2014 y luego utilizar esta información en la estimación de los costos económicos por la pérdida de productividad debida a defunciones cardiovasculares asociadas con la inactividad física.

## MATERIALES Y MÉTODOS

### Cálculo de las fracciones de riesgo poblacional atribuibles (FAP) a la inactividad física.

Para este cálculo se utilizaron como insumos los riesgos relativos (RR) estimados en un metanálisis (14), junto con la proporción de prevalencia de este factor de riesgo obtenida de los microdatos de la ENFR del año 2013. La FAP puede ser expresada matemáticamente como:

$$FAP=\frac{P(\text{D})-\sum_{C}P(D/C,E)P(C)}{P(D)}[1]$$

Donde *P*(*D*) es la probabilidad promedio de la enfermedad en una población que contiene expuestos y no expuestos, y Σ_*c*_ *P*(*D* | *C*, *E*) *P* (*C*) representa la probabilidad marginal condicional de enfermedad en ausencia de exposición promediada a lo largo de los estratos de otros factores de riesgo (15). Para estimar las FAP se han utilizado varias fórmulas y en este caso se empleó la siguiente ecuación para distintas categorías de exposición como aproximación empírica a la expresión anterior:

$$FAP=1-\frac{1}{\sum_{i=0}^{k}\left(p_{i}RR_{i}\right)}[2]$$

donde *p*_*i*_ es la proporción de la población perteneciente al nivel de exposición *i*, y *RR*_*i*_, el riesgo relativo en el nivel de exposición *i*.

### Estimación de la mortalidad atribuible a las enfermedades cardiovasculares asociadas con la inactividad física.

Se computó mediante la ecuación 3 como el producto entre las FAP y el número de muertes originadas por las enfermedades cardiovasculares asociadas:

$$MA_{s,e,naf}=\$Muertes{\$}_{s,e}\cdot FAP_{s,e,\mathrm{maf}}[3]$$

donde la MA al factor de riesgo es el producto del número de defunciones estratificado por grupos de edad (*e*) y por sexo (*s*), por las FAP estratificadas por grupos de edad, sexo y nivel de exposición al factor de riesgo (nivel de actividad física). Dado que en el metanálisis no se desagregan las causas de muerte más allá de enfermedades cardiovasculares o accidentes cerebrovasculares, no es posible desagregar las estimaciones de los costos económicos por enfermedad. Por lo tanto, para la estimación de los costos se tuvieron en cuenta las enfermedades hipertensivas (I10-I15), las enfermedades isquémicas del corazón (I20-I25), otras formas de enfermedad del corazón (I30-I52), y las enfermedades cerebrovasculares (I60-I69) según la Clasificación Internacional de Enfermedades 10 edición y en relación con las defunciones por enfermedad contenidas en el informe de estadísticas vitales para 2014 de la Dirección de Estadísticas e Información de Salud (16).

### Valoración: productividad perdida por defunciones cardiovasculares atribuibles a la escasa actividad física.

Se realizó una valoración del valor estadístico de una vida (VEV) siguiendo el enfoque del capital humano, donde la vida estadística se estima por la productividad perdida por muerte prematura:

$$VPIF_{i}=\sum_{j=i}^{99}p{\left(\mathrm{Viva}\right)}_{i}^{j}$$

$${\mathrm{Ingreso}}_{j}\cdot {\left(1+g\right)}^{j-i}\cdot {\left(\frac{1}{1+r}\right)}^{j-i}[4]$$

El VEV se puede aproximar con la fórmula actuarial propuesta en la ecuación 4, en donde se estima la productividad perdida por una muerte prematura con los ingresos de fuente laboral que percibe un individuo descontándolos para obtener el valor presente de los ingresos futuros (VPIF). En esta ecuación *p*(*viva*) es la probabilidad de que la persona de la edad *i* esté viva a la edad *j*, *Ingreso* es el ingreso medio laboral de las personas de edad *j*, *g*, la tasa de crecimiento del ingreso medio, y *r*, la tasa de descuento. La tasa de crecimiento del ingreso medio de fuente laboral se calculó como el valor promedio de crecimiento del PIB per cápita desde 1983 (vuelta a la democracia) hasta 2014. Se optó por adoptar una tasa de descuento media del 5% por considerarse moderada. La probabilidad de sobrevida se estimó utilizando las tablas actuariales de Grushka (17). Para calcular la probabilidad de sobrevivir de un individuo de edad *i* a la edad *i* + *n* se utilizó la siguiente fórmula:

$$p{\left(viva\right)}_{i}^{j}=\frac{1(i+n)}{1(i)}[5]$$

El ingreso total medio por edad es el ingreso que habría percibido el individuo si hubiera sobrevivido. Para su cálculo, se utilizaron los microdatos de la Encuesta Permanente de Hogares (EPH) del primer trimestre de 2014, de donde solamente se tomaron los ingresos laborales para todos los aglomerados ponderados por su factor de expansión. Las variables que se utilizaron para calcular el ingreso medio de la EPH del primer trimestre de 2014 fueron P21 (ingresos laborales), Pondera (factor de expansión) y CH06 (edades) promediadas para todo el país. Sin embargo, no se diferenció el ingreso por género debido a que, si se hiciera, habría una diferencia muy marcada entre las remuneraciones por no considerar la producción implícita del hogar, sumada a una menor participación de las mujeres en el mercado laboral y menores sueldos promedio.

Posteriormente, se realizó un análisis de sensibilidad proponiendo distintos escenarios, con el objetivo de evaluar cómo se modifican los outputs (VEV o VPIF) cuando varían los inputs (tasa de crecimiento del producto promedio, tasa de descuento) entre los grupos de edad. Para realizar este análisis, se hizo una simulación de Montecarlo con 20 000 iteraciones con el programa Simula 4.0 para Excel. En ella se utilizó una distribución triangular que permitió reflejar distintos escenarios respecto de la variabilidad de los valores elegidos. En cuanto a los inputs, se estableció para la tasa de crecimiento del producto un límite inferior de 1% y un máximo de 7% (≈ del promedio del período 2003–2014). Respecto de la tasa de descuento, se fijaron un límite inferior de 3% y un límite superior de 7%, tal como recomienda Harrison para los análisis de sensibilidad en estos tipos de estudios (18).

### Costos económicos asociados con las enfermedades cardiovasculares atribuibles a la inactividad física.

Los costos económicos por muerte prematura asociados con la pérdida de productividad atribuible a la inactividad física pueden aproximarse mediante la siguiente ecuación:

$$CMA_{s,e,\mathrm{mat}}=MA_{s,e,mf}\cdot VPIF_{e}\;[6]$$

donde *CMA*_*s*,*e*,*naf*_ es el costo económico por muerte prematura asociado con la pérdida de productividad atribuible a la escasa actividad física estratificado por grupo de edad (*e*), sexo (*s*) y nivel de exposición al factor de riesgo, y *VPIF*_*e*_, el valor presente de los ingresos futuros o VEV por grupo de edad.

Para que los valores monetarios de los pesos argentinos pudieran compararse a escala internacional, se convirtieron a dólares internacionales (I$) empleando el factor de conversión de paridad de poder adquisitivo de 2014 equivalente a 4,65 pesos argentinos (19).

## RESULTADOS

**FIGURA 1.**
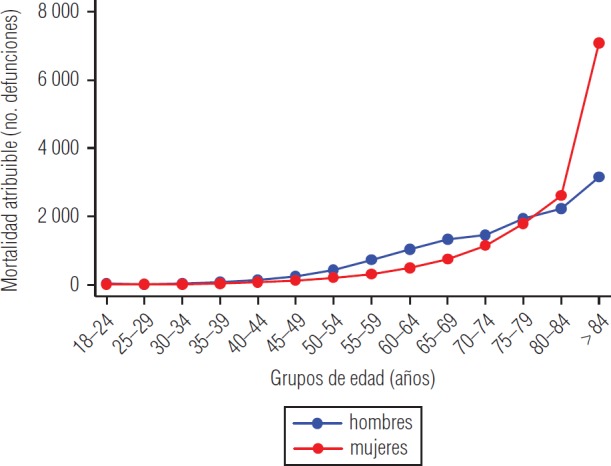
Mortalidad atribuible a la inactividad física por grupos de edad y sexo, Argentina, 2014

En la figura 1 se presenta la mortalidad calculada para individuos con un nivel de actividad física bajo y estratificada por sexo y edad. En ella se observa que tras el grupo de 35–39 años comienza una brecha entre las dos curvas que llega al punto máximo en el grupo de 65–69 años como consecuencia de la gran cantidad de defunciones que se observa para esa franja de edad en los hombres. Esta tendencia se invierte después del grupo de 75–79 años en el cual la curva de mortalidad de las mujeres se dispara porque presentan un mayor número de defunciones en los últimos grupos de edad y porque la brecha de prevalencia de inactividad física alanza su punto máximo.

***Fuente:*** elaboración propia a partir de la mortalidad atribuible estimada por grupos de edad y sexo.

**FIGURA 2.**
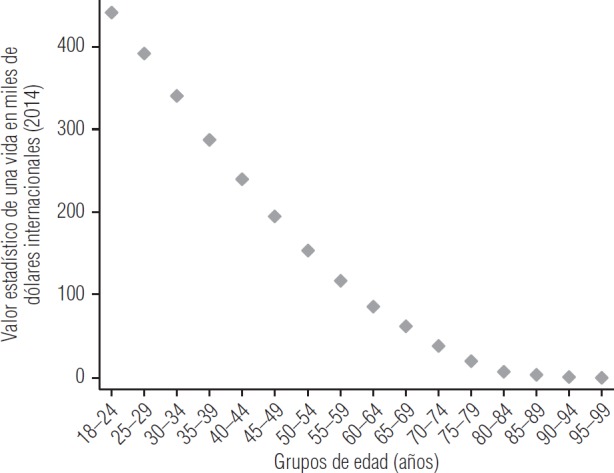
Valor estadístico de una vida por grupos de edad, Argentina, 2014

***Fuente:*** elaboración propia a partir del valor estadístico de una vida estimado por grupo de edad.

***Nota:*** las cifras se presentan en miles de dólares internacionales (I$). Para la conversión de pesos argentinos a dólares internacionales se utilizó el factor de conversión de paridad de poder adquisitivo del 2014 (I$ 1 = $ 4,65).

La figura 2 ilustra el VEV sobre la base del primer trimestre de la EPH 2014. Puede observarse que la muerte prematura de los individuos de 18 a 24 años es la más costosa (VEV = I$ 441 005). Si se estratifica este grupo, el mayor costo por muerte prematura lo presentan los individuos de 18 años (VEV = I$ 469 016). Por otro lado, en los adultos maduros los VEV son menores por tener menos años de vida por delante y también porque su probabilidad de sobrevida baja junto con sus ingresos esperados.

**CUADRO 1.** Análisis de sensibilidad del valor estadístico de una vida por grupos de edad, Argentina, 2014

Grupo de edad (años)	Máximo	Media	Mínimo	Desvío sobre la media
	(I$^a^)	(I$)	(I$)	(%)
18–24	1 282	322,9	138,2	40,9
25–29	1 034	305,2	144,2	35,5
30–34	838,5	282,6	144,4	31,2
35–39	671	254,1	138,3	27,7
40–44	525,1	222,7	128,6	24,4
45–49	402,1	190,8	117,2	21,2
50–54	305,6	160,6	104,5	18,3
55–59	223,2	130	89,7	15,5
60–64	162,8	105	77,2	12,6
65–69	107,7	77	60,7	9,6
70–74	66,7	52,4	44,1	6,9
75–79	25,9	21,5	18,9	5,2
80–84	7,2	5,8	5	6
> 84	1,4	0,9	0,7	12,1
Promedio simple (%)	403,8	152,3	86,6	19,1
Tasa de crecimiento del producto (%)	6,9	3,3	1	39,3
Tasa de descuento (%)	6,9	4,9	3	16,3

***Fuente:*** elaboración propia a partir de los resultados presentados.

***Nota:*** las cifras se presentan en miles de dólares internacionales (I$). Para la conversión de pesos argentinos a dólares internacionales se utilizó el factor de conversión de paridad de poder adquisitivo de 2014 (I$ 1 = $ 4,65).

El cuadro 1 incluye los resultados del análisis de sensibilidad para los VEV por grupo de edad respecto a los diferentes escenarios simulados. En función de estas simulaciones, se observa que los VEV varían desde un promedio mínimo de I$ 86 590, con una media de I$ 152 292 y un promedio máximo de I$ 403 844.

**FIGURA 3.**
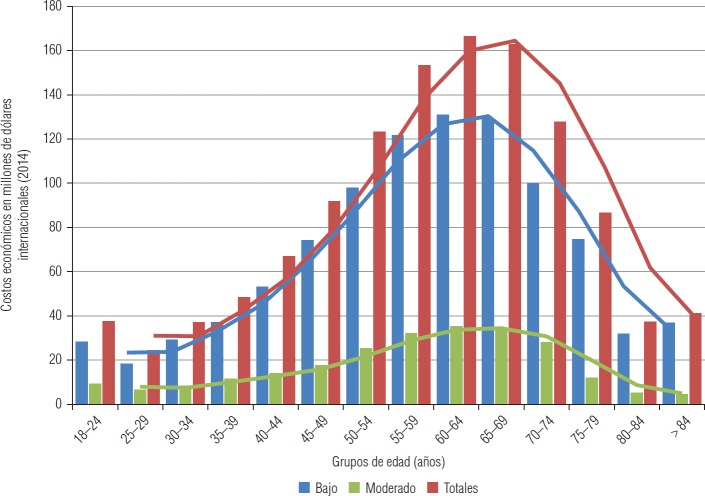
Costos económicos por muerte prematura asociados con la pérdida de productividad atribuibles a la inactividad física por enfermedades cardiovasculares por grupos de edad y nivel de actividad física, Argentina, 2014

La figura 3 muestra los costos económicos por muerte prematura asociados con la pérdida de productividad atribuibles a la inactividad física por enfermedades cardiovasculares y accidentes cerebrovasculares distribuidos por niveles de actividad física (bajo, moderado) y grupos de edad. El desplazamiento de las curvas se produce por la interacción entre los valores de los RR, de la proporción de prevalencia del factor de riesgo y de los VEV, que varían entre los individuos más jóvenes (que tienen un VEV muy alto y un RR bajo aun cuando no realicen actividad física) y los adultos mayores. Sin embargo, en este gráfico sólo se logra captar las variaciones de los valores de los VEV a diferentes edades, lo que no sucede con los RR (más allá de las diferencias por nivel de actividad física), ya que éstos no se estratificaron por edad. En el nivel bajo, se observa que desde el grupo de 25–29 años los costos aumentan hasta alcanzar su máximo en el grupo de 60–64 años (I$ 130, 8 millones). Si bien este grupo no presenta el valor máximo de las mortalidades calculadas, su VEV es sustancialmente mayor que el de los siguientes grupos de edad. Tras alcanzar este máximo, el VEV del siguiente grupo de edad (65–69 años) disminuye levemente respecto al grupo anterior, descenso que va ascendiendo en los grupos posteriores. En el nivel moderado, al igual que en el anterior, el punto de inflexión se produce en el grupo de 60–64 años (I$ 34,9 millones). Finalmente, los costos decrecen por la marcada disminución de los VEV hasta el grupo de 80–84 años.

Como valoración total de los costos por sexo se obtuvo una suma de I$ 752,5 millones para los hombres y de I$ 444,5 millones para las mujeres. Esta marcada diferencia se produce a causa principalmente de que las mujeres presentan un número mayor de MA en los grupos de edad más avanzada, lo cual se puede verificar en la figura 1, donde la cantidad de MA en las mujeres se produce en el grupo de edad de las mayores de 75–79 años, en el cual los VEV son mucho menores.

El cuadro 2 contiene los valores del análisis de sensibilidad de los VEV extrapolados para computar los costos económicos asociados con la pérdida de productividad por muerte prematura atribuibles a la inactividad física por enfermedades cardiovasculares y accidentes cerebrovasculares en tres escenarios diferentes (mínimo, medio y máximo). En el primer escenario (g = 1%; r = 3%), la suma de los costos del nivel bajo y moderado produce para los hombres un total de I$ 586,6 millones y para las mujeres, I$ 337,7 millones. En el segundo escenario (g = 3,34%; r = 5%), en cambio, se presenta una suma de I$ 821,3 millones para los hombres y de I$ 465.2 millones para las mujeres. En el tercer escenario (g = 7%; r = 7%), la suma generada para los hombres es de I$ 1 436,4 millones y para las mujeres, de I$ 801,1 millones.

En función de lo anterior, se estimó que las pérdidas económicas variaron desde un mínimo de I$ 924,3 millones, con un valor medio de I$ 1 286,5 millones, hasta un máximo de I$ 2 237,5 millones. El valor mínimo representa cerca de 0,61% del PIB (a precios de 2014, I$ 21,5 per cápita), el valor medio, 0,85% (I$ 29,9 per cápita) y el valor máximo, 1,48% (I$ 52 per cápita).

## DISCUSIÓN

La actividad física insuficiente se ha convertido desde hace varios años en un tema preocupante a escala internacional. En 2014, cerca de 23% de adultos de 18 años o más de edad y 81% de los adolescentes (11 a 17 años) no eran suficientemente activos (20). Por ello, este trabajo se concentró en estimar la mortalidad y los costos económicos producidos por enfermedades cardiovasculares asociados con la inactividad física en Argentina.

***Fuente:*** elaboración propia a partir del costo económico por mortalidad atribuible estimado por grupo de edad y nivel de actividad física**.**

***Nota:*** las cifras se presentan en millones de dólares internacionales (I$). Para la conversión de pesos argentinos a dólares internacionales se utilizó el factor de conversión de paridad de poder adquisitivo de 2014 (I$ 1 = $ 4,65).

**CUADRO 2.** Comparación de escenarios sobre los costos económicos asociados con la pérdida de productividad por muerte prematura atribuibles a la inactividad física por grupos de edad, sexo y nivel de actividad física (bajo, moderado), Argentina, 2014

Grupo de edad	Mínimo (g = 1%; r = 3%)				Escenario Medio (g = 3,34%; r = 5%)				Máximo (g = 7%; r = 7%)			
Sexo	Hombre		Mujer		Hombre		Mujer		Hombre		Mujer	
	Nivel de actividad física				Nivel de actividad física				Nivel de actividad física			
	Bajo (I$^a^)	Moderado (I$)	Bajo (I$)	Moderado (I$)	Bajo (I$)	Moderado (I$)	Bajo (I$)	Moderado (I$)	Bajo (I$)	Moderado (I$)	Bajo (I$)	Moderado (I$)
18–24	5,2	1,7	3,5	1,1	12,3	4	8,2	2,6	49	15,9	32,6	10,3
25–29	4,1	1,3	2,4	0,9	8,8	2,8	5,2	2	29,8	9,6	17,7	6,9
30–34	7,4	2,1	4,6	1,2	14,6	4,2	9,1	2,4	43,4	12,5	27,1	7,1
35–39	10,9	3,2	6,7	2,1	20,1	5,9	12,3	4	53,2	15,7	32,5	10,6
40–44	18,4	4,1	10,1	3,1	31,9	7,2	17,5	5,3	75,3	17	41,4	12,7
45–49	30,4	7	13,8	3,5	49,6	11,4	22,5	5,8	104,5	24,1	47,6	12,2
50–54	45	12,2	21,4	5	69,2	18,8	33	7,7	131,8	35,8	62,8	14,6
55–59	65,5	17	27,9	7,2	95	24,7	40,4	10,5	163,1	42,5	69,4	18,1
60–64	80,2	21,7	38,3	9,9	109	29,5	52,1	13,5	169	45,8	80,8	20,9
65–69	81,7	23	45,7	9,7	103,6	29,2	58	12,3	144,9	40,8	81,1	17,3
70–74	64,5	21,7	50,4	9,9	76,6	25,8	59,9	11,8	97,6	32,8	76,3	15
75–79	36,7	6,4	33,9	4,6	41,7	7,3	38,6	5,3	50,2	8,8	46,4	6,4
80–84	11,2	1,5	13,1	2,3	13	1,7	15,1	2,6	16,1	2,1	18,8	3,3
>84	2,2	0,3	4,9	0,5	3	0,4	6,8	0,7	4,5	0,6	10,1	1,1
Total^b^	463,4	123,2	276,7	61	648,4	172,9	378,7	86,5	1 132,4	304	644,6	156,5
Promedio simple^c^	33,1	8,8	19,8	4,4	46,3	12,3	27,1	6,2	80,9	21,7	46	11,2
Total^d^	586,6		337,7		821,3		465,2		1 436,4		801,1	

***Fuente:*** elaboración propia a partir del análisis de sensibilidad del valor estadístico de la vida (VEV) y de la mortalidad atribuible (MA) estimada por nivel de actividad física, grupo de edad y sexo. En el escenario mínimo se utilizó una tasa de crecimiento (g) de 1 % y una tasa de descuento (r) de 3 %. En el escenario medio se utilizó una tasa de crecimiento promedio de 3,34 % y una tasa de descuento de 5 %. Para el escenario máximo se empleó una tasa de crecimiento del producto de 7 % con una tasa de descuento de 7 %.

a Las cifras se presentan en millones de dólares internacionales (I$). Para la conversión de pesos argentinos a dólares internacionales se utilizó el factor de conversión de paridad de poder adquisitivo de 2014 (I$ 1 = $ 4,65).

b El total se calculó para todos los niveles estratificando por sexo.

c El valor promedio simple se calculó para todos los niveles de actividad física estratificando por sexo.

d El total se calculó sumando los niveles bajo y moderado sin estratificar por sexo.

Los resultados obtenidos pueden compararse con los de otros estudios realizados en Argentina que han utilizado el mismo método con otros factores de riesgo. Por ejemplo, Conte Grand estimó que el costo económico del tabaco para 2003 fue 0,14% del PIB (21), Avendaño, que para 2007 el costo de la productividad perdida por muertes asociadas con el sida ascendió a 0,40% del PIB (13), y el Observatorio Argentino de Drogas calculó el costo del abuso de sustancias psicoactivas: para el consumo de tabaco la carga económica fue 0,91%, para el consumo de alcohol, 1,07%, y para el consumo de drogas legales e ilegales 2,93% del PIB (12).

Los resultados indican una importante carga de mortalidad y costos. Por lo tanto, el desarrollo de políticas públicas dirigidas a reducir el sedentarismo se debe incorporar en la agenda de los responsables políticos. Para ello, es indispensable conocer el horizonte temporal en que se generarían los beneficios esperados por una intervención estatal. En este sentido, es importante mencionar que existe amplia evidencia científica que demuestra una relación causal entre mejoras en los perfiles cardiovasculares de los individuos tras implantar un programa de actividad física a corto plazo (22–25).

También es útil preguntarse si la tendencia de este factor de riesgo es inducida por fallos de mercado o si el sedentarismo condiciona a las personas inactivas a estimar mayores costos que las personas más activas, haciendo caer a las primeras en un círculo vicioso. Por ejemplo, unos investigadores analizaron la influencia de las experiencias pasadas en actividades físicas sobre el cálculo de costo-beneficio que realizan los individuos. De su análisis se deriva que los costos de hacer ejercicio son significativamente más altos para los individuos con menos experiencia en hacer ejercicio que para los que tienen más experiencia, porque los primeros podrían haber tenido una experiencia menos positiva con el ejercicio y más positiva en las actividades de ocio no activo (26).

Otros investigadores han generado un debate sobre la intervención del Estado con la obesidad y se han preguntado si el seguro de salud produce problemas de riesgo moral que puedan desencadenar obesidad, si los individuos obesos podrían causar externalidades negativas sobre las demás personas no obesas o si la obesidad es generada en parte por un problema de autocontrol o de información asimétrica sobre los riesgos de la misma. Evidentemente, algunos de estos problemas pueden ampliarse al debate sobre la intervención pública en el sedentarismo, aunque, al igual que la obesidad, constituye un problema social complejo en el cual la justificación de una intervención pública no tiene una única respuesta (27).

En cuanto a las limitaciones de este estudio debe mencionarse que no se tienen en cuenta los costos generados por realizar ejercicio físico, ni tampoco los costos directos por atención médica, ni las pérdidas económicas generadas por los individuos que quedan excluidos del mercado laboral por alguna discapacidad derivada de las enfermedades cardiovasculares.

No obstante, la importancia de este trabajo radica en que es el primero en que se estiman la mortalidad y los costos por defunciones cardiovasculares atribuibles a la inactividad física en Argentina, lo cual es relevante por dos razones. Por un lado, sienta un precedente para llevar a cabo evaluaciones posteriores y ampliar la investigación a análisis de costo-beneficio. Por otro, permite a los responsables políticos evaluar cuál es la situación a escala nacional. Otro punto importante se refiere a su vertiente metodológica. El enfoque elegido para calcular los costos económicos fue el del VEV, una medida que describe la tasa marginal de sustitución (o compensación) entre el riesgo de mortalidad y un determinado valor monetario en un período establecido (28). Si bien este enfoque no está exento de problemas (29), su uso se ha extendido ampliamente y constituye el principal parámetro económico de la lucha a favor de la reducción del riesgo de mortalidad (30), lo que permite comparar los resultados obtenidos a escala internacional.

En conclusión, los resultados de este estudio sugieren que la inactividad física presenta una importante carga económica y de mortalidad para la población de Argentina y, en consecuencia, se recomienda tanto desarrollar líneas de investigación para explorar los factores que afectan a la práctica de actividad física, como adoptar medidas eficientes destinadas a la reducción de este factor de riesgo.

### Agradecimiento.

Los autores agradecen los comentarios y sugerencias realizados por Mariano Rabassa (Universidad Católica de Argentina) y Mariana Conte Grand (Universidad del CEMA).

### Financiación.

Este estudio no recibió financiación.

### Declaración.

Las opiniones expresadas por los autores son de su exclusiva responsabilidad y no reflejan necesariamente los criterios ni la política de la Organización Panamericana de la Salud o de la *RPSP/PAJPH*.
